# Supplementary figures and images for: Transcriptome profiling of anthocyanin-related genes reveals effects of light intensity on anthocyanin biosynthesis in red leaf lettuce
Source: PeerJ. 2018 Apr 13;6:e4607. doi: 10.7717/peerj.4607 (PMC5900932; doi:10.7717/peerj.4607)

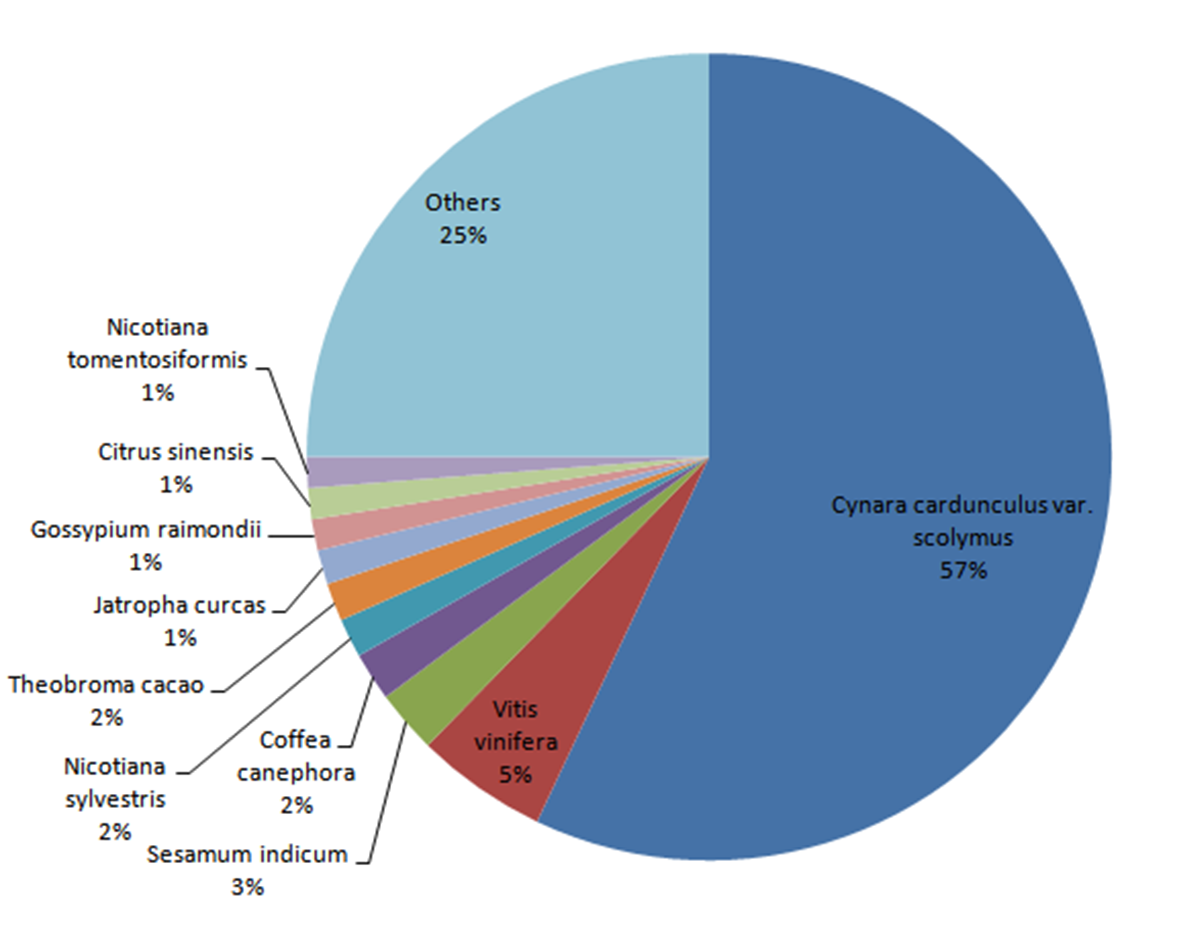

Supplement: Supplemental Information 1 — This figure shows the species distribution of unigene BLASTX results against the nr protein database with a cutoff E value <10−5 and the proportions of each species. Different colors represent different species. [file peerj-06-4607-s001.png]

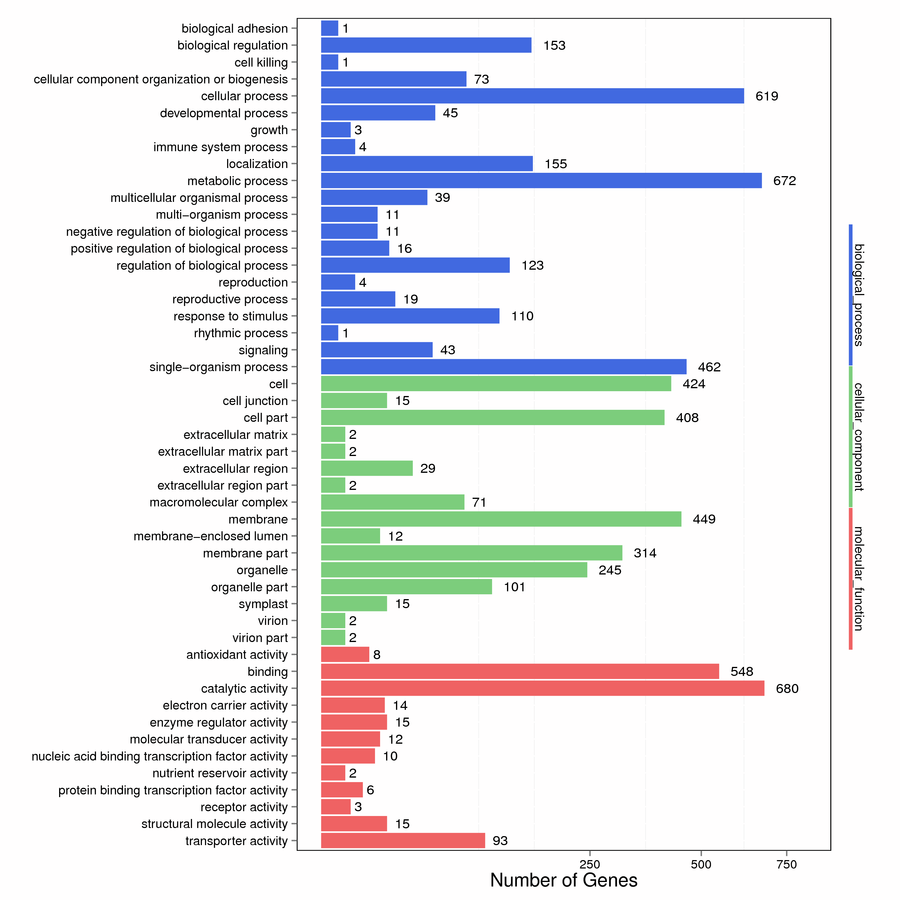

Supplement: Supplemental Information 2 — Unigenes were annotated in three categories: cellular components, molecular functions, biological processes. [file peerj-06-4607-s002.png]

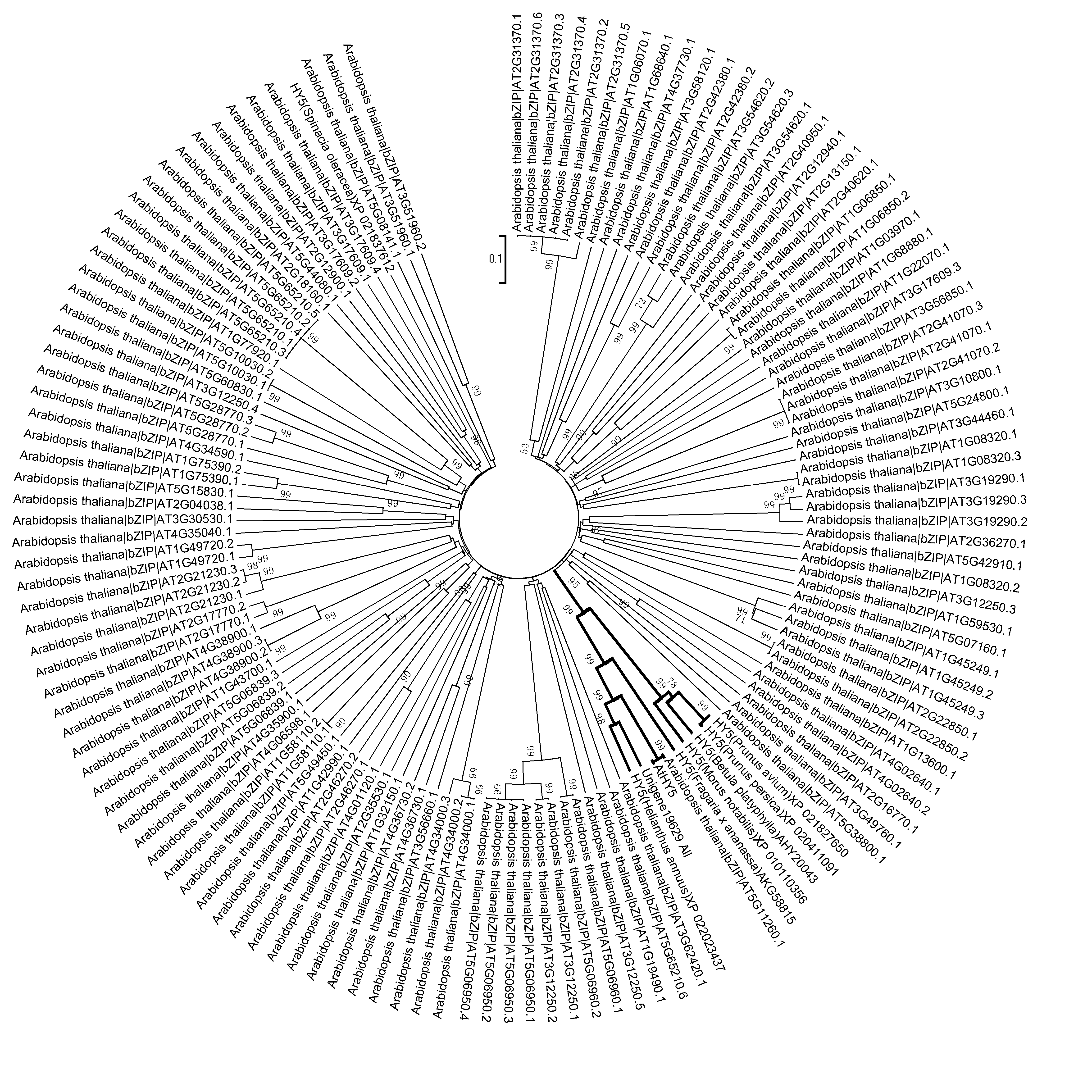

Supplement: Supplemental Information 3 — Arabidopsis bZIPs were down load from PlantTFDB database (http://planttfdb.cbi.pku.edu.cn/). [file peerj-06-4607-s003.png]
