# Supplementary material for: Transcriptome profiling of anthocyanin-related genes reveals effects of light intensity on anthocyanin biosynthesis in red leaf lettuce
Source: PeerJ. 2018 Apr 13;6:e4607. doi: 10.7717/peerj.4607 (PMC5900932; doi:10.7717/peerj.4607)
Supplement: Supplemental Information 4 [file peerj-06-4607-s004.docx]

Table S1 Primers used for qRT-PCR

| **Primer name** | **sequence（5’-3’）** | **Product length（bp）** |
| --- | --- | --- |
| Actin F | AGGGCAGTGTTTCCTAGTATTGTTG | 106 |
| Actin R | CTCTTTTGGATTGTGCCTCATCT |  |
| 3GT-1 F | CAAAGGTAATTCAGTTGGGC | 173 |
| 3GT-1 R | TTGCAACAACGGTGGCTTTG |  |
| 3TG-2 F | CAAAGGTAATTCATTAGCGCC | 174 |
| 3TG-2 R | ACGAGAACAACGCTGGCTTT |  |
| ANS F | GGAATCTCCGACGACCTCAT | 168 |
| ANS R | GTCTTCCCATTCGAGTTGCC |  |
| bHLH F | GCAACATCTATGGCTCACGG | 153 |
| bHLH R | CCCTCCTCCACCTTTTCAGT |  |
| CHI F | TGGTCCCTTCGAGAAACTGA | 124 |
| CHI R | TGCATCTGTGTAGGTTCCGT |  |
| CHS-1 F | TTCTGACCCATTGCCCGAT | 119 |
| CHS-1 R | ATGTAAGCCCGACCTCACG |  |
| CHS-2 F | GTGCTCGTGTTCTTGTGGTT | 156 |
| CHS-2 R | GCCGTTCAATCGTCAAGTCA |  |
| DFR F | CACTCCCTCATTCCCTCCAA | 153 |
| DFR R | CCTTCGGCTTTTGGGTTCTC |  |
| F3H F | GGTGTTAATGGGTTTGGCGT | 146 |
| F3H R | TGAGATCGGGTTGAGGACAC |  |
| F3'H F | CCACGGTCAAATTAGGCCAG | 158 |
| F3'H R | TCCGGCTAACACCATCAGTT |  |
| GST F | CCACAGATCCGAACCCGTA | 141 |
| GST R | ACTCCCATGGCTTCTCTGG |  |
| HY5 F | TGCTGATAGAGAAAGCAAGCG | 151 |
| HY5 R | CAAACGTTCTTCAAGCTCCGA |  |
| MATE F | CCGTTTGCATGGGTGTCAA | 111 |
| MATE R | CTGCGGCTTTAGGGTTTCC |  |
| MYB-1 F | AACCCTCGTTCCAAACAACC | 132 |
| MYB-1 R | GTTGTCACCCATGCACTCG |  |
| MYB-2 F | TGCGGGAAGAATACCAGGG | 152 |
| MYB-2 R | AGCTTCGTGGTTGAGGTTT |  |
| MYB-3 F | ACTGTTACAAGACGCTCCAGT | 142 |
| MYB-3 R | GCCACCTTCATTCGACGATC |  |
| MYB-4 F | CATTGACACCGCATGCAGATA | 123 |
| MYB-4 R | CATGCCTTTCCACATCCTCAG |  |
| MYB-5 F | TCCCCACCAATAGCAGTGAAT | 125 |
| MYB-5 R | CCAAACTCATCGCTAACACCA |  |
